# Immune Cells in the Uterine Remodeling: Are They the Target of Endocrine Disrupting Chemicals?

**DOI:** 10.3389/fimmu.2020.00246

**Published:** 2020-02-19

**Authors:** Nicole Meyer, Ana Claudia Zenclussen

**Affiliations:** Experimental Obstetrics and Gynecology, Medical Faculty, Otto-von-Guericke University, Magdeburg, Germany

**Keywords:** uterine remodeling, immune cells, pregnancy, pregnancy pathologies, endocrine disrupting chemicals, fetal development, maternal health

## Abstract

Sufficient uterine remodeling is essential for fetal survival and development. Pathologies related to poor remodeling have a negative impact on maternal and fetal health even years after birth. Research of the last decades yielded excellent studies demonstrating the key role of immune cells in the remodeling processes. This review summarizes the current knowledge about the relevance of immune cells for uterine remodeling during pregnancy and further discusses immunomodulatory effects of man-made endocrine disrupting chemicals on immune cells.

## Introduction

In the last years it has become clear that a healthy intrauterine environment builds the fundament of adult health (1). The efficient remodeling of tissue and vasculature within the uterus during the menstrual cycle and pregnancy is a basic requirement for a healthy intrauterine environment (2, 3). Several studies of the last decades have revealed the relevance of maternal immune cells, specifically uterine natural killer cells (uNKs), macrophages, and T cells but also fetal trophoblast cells for the remodeling process during pregnancy (4). Research within the field has experienced significant progress in recent years and provide new insights. Specifically, there is an increased knowledge about the importance of uterine mast cells (uMCs) for the vascular remodeling process during pregnancy. Equally important, a negative influence of man-made endocrine disrupting chemicals (EDCs) on reproductive health emerge within the literature in recent years, leading to further relevant health questions that need answers, opening new challenging research fields.

## Cyclic Endometrial Remodeling

The endometrium of women in reproductive age undergoes cyclic tissue remodeling each month. The menstrual cycle aims to prepare the endometrium for implantation. The activity of the ovarian steroid hormones estrogen and progesterone but also various matrix metalloproteinases (MMPs) and their inhibitors, named tissue inhibitors of MMP (TIMP), regulate the endometrial changes and tissue breakdown during menstruation (5).

The endometrium consists of two layers, the functional layer that is shed with each menstruation, and the basalis layer containing progenitor cells, able to regenerate the functional layer. The proliferative phase of the menstrual cycle is maintained by the hormonal influence of estrogen. Epithelial and stromal cells undergo mitosis and growth, glands increase in length and the extracellular matrix (ECM) expands. Endometrium thickness increases from around 4.5 to 10 mm. The secretory phase of the menstrual cycle is mainly maintained by the effects of progesterone. High levels of progesterone, secreted by the *corpus luteum*, are responsible for endometrial receptivity after ovulation. Progesterone inhibits the endometrial epithelial mitosis and proliferation, induces the appearance of vacuoles, stromal fibroblast changes, and alterations in ECM that are necessary for the receptivity of the endometrium and subsequently, for the blastocyst attachment (6).

If the fertilization of the oocyte does not take place, the *corpus luteum* degrades, and the declining progesterone levels cause a local inflammatory response in the endometrium, involving edema and infiltration of specialized maternal immune cells into the stroma (3), predominantly uNKs, macrophages, uMCs, neutrophils, dendritic cells (DCs), and T cells (7). The presence of chemokines in this milieu is important for leukocyte recruitment (8). Macrophages and neutrophils that are recruited to the site represent the microbial protection mechanism while the epithelial barrier is disrupted. MC proteases transform MMP precursors into their active form (9). MMPs in turn degrade the ECM and destruct the tissue. The final result is shedding of the endometrial functional layer, and thereby two-third of the endometrium, during the menstrual phase of each cycle (6). As antigen-presenting cells, DCs and macrophages clear the cellular debris from the uterine cavity. Regulatory T cells (Tregs) control all these processes and maintain the immune balance to avoid an exacerbated inflammatory response (9). Disturbances in endometrial immune cell number or function have been found to contribute to heavy menstrual bleeding or endometriosis (7). Menstruation occurs in human, primates, elephants, and fruit bats. Non-menstruating species show a considerable remodeling and reabsorption of the endometrium (5). A subsequently regeneration, including tissue and vascular repair, growth, and angiogenesis facilitates the receptivity of the endometrium for implantation in the next cycle (10). Also here immune cells play a key role by releasing regulatory molecules stimulating the endometrial repair mechanisms (7).

## Uterine Remodeling During Healthy Pregnancy

In non-menstruating mammals, decidualization begins with the implantation process. In contrast, in menstruating species, decidualization occurs prior to implantation and is postulated to be a mechanism to protect the mother from the invasiveness of embryonic trophoblasts. A successful implantation process is followed by several tissue and vascular adaptions. The most important tissue adaption in this regard is the formation and growth of a new transient organ, the placenta. Maternal blood is delivered to the intervillous space of the placenta via the aorta, the uterine artery, the arcuate artery, radial arteries, and spiral arteries (SA), listing from large to small vessels. In response to the altered hemodynamic demands resulting from an increased uterine blood flow during pregnancy, there is the need of a physiological remodeling of the uterine vasculature. The remodeling process starts in the smaller vessels, the SAs, proximal to the sites of placentation and proceeds to the larger, upstream vessels (11).

Many studies focused and still focus on the remodeling of SAs. The helically wounded arteries build the last branch of the uterine artery. SAs transport maternal blood to the intervillous space of the placenta, where the blood enters in direct contact with fetal tissue, for an effective exchange of nutrients and gases (12). During pregnancy, the thick-walled, high resistance vessels transform into thin-walled low resistance vessels by losing several vascular smooth muscle cell (VSMC) layers of the arterial wall (13, 14).

VSMCs are aligned in a circumference in the medial layer of the arterial wall. For maintaining the vascular tone, VSMCs usually acquire a quiescent, contractile phenotype. The contractile phenotype is characterized by high expression of contractility markers and low proliferative or migratory activity. An enormous plasticity enables VSMCs to change their morphology during pregnancy and consequently their functionality changes as well. Expression patterns change leading to increased proliferation, migration, and synthetic capacity (15). These parameters, together with a low expression of contractility markers, are characteristic for the synthetic phenotype of VSMCs. VSMCs can change their expression pattern due to vascular injury or changing hemodynamic demands (16) in response to various stimuli, ligand-receptors interactions, and environmental signals (17). The ECM compounds collagen, elastin, and proteoglycans facilitate a contractile VSMC phenotype. In contrast, high presence of fibronectin favors the shift into a synthetic VSMC phenotype (18). A phenotype switch from the contractile to synthetic VSMCs is associated with a changed protein and receptor expression that modify the binding specificity to the ECM, and an increased VSMC migration (18, 19), that in turn is important for an efficient SA remodeling process (20, 21).

A propervascular remodeling is important for fetal survival, development, and growth for the following reasons. Firstly, an enlarged arterial diameter reduces the velocity of the blood stream (14) and prevents disturbance of the sensitive fetal villi containing fetal blood capillaries. Secondly, due to the loss of muscular VSMC layers, SAs completely lose their contractile ability, preventing an interruption or reduction of the blood stream to the placenta that would be incompatible with fetal survival (22, 23).

Both, maternal and fetal cells contribute to the uterine remodeling process. In preparation for the remodeling process, maternal immune cells release factors that induce the degradation the ECM and directly or indirectly induce apoptosis or a phenotypic switch of VSMCs leading to higher migration (19, 21). Subsequently, fetal trophoblast can invade into the SAs and replace VSMCs.

## Immune Cell Function in Uterine Remodeling During Pregnancy

### Interaction of Stromal Cells, Immune Cells, and Trophoblasts

The feto-maternal interface consist of decidual stromal cells, immune cells, and trophoblasts. Differentiation of stromal cells within the decidualization process is a pre-requirement for a successful implantation (24).

Stromal cells express integrins, important for the interaction of the blastocyst with the decidua during the implantation process (25). Additionally stromal cells produce chemokines that attract immune cells to the uterus. Interesting examples are Mcp-1, RANTES, or CXCL8 that attract monocytes and macrophages (26, 27), CXCL10 and CXCL11 that attract uNKs (28), and IL-15 and CXCL12 that act as chemoattractants for pNKs (29, 30). Carlino et al. showed that the migratory ability of NKs from pregnant women was higher compared to those of non-pregnant women. The process could be enhanced by progesterone, that upregulated chemokine production by stromal cells (30). Decidual stromal cells are localized near SAs and support their remodeling by the secretion of MMP 2, 7, and 9. These factors are able to promote the disruption of VSMS layers (31) prior to the invasion of extravillous trophoblasts (EVTs). Several studies let assume an association between stromal cell dysfunction and reproductive disorders like endometriosis (32), implantation failure (33) or recurrent pregnancy loss (34).

EVTs are important key players in the vascular remodeling during pregnancy. EVTs arise from the cytotrophoblast in the anchoring villi of the placenta and invade into the maternal SA, thereby replacing VSMCs and the endothelium. By using a three-dimensional bioprinted placenta model, Kuo et al. performed impressive trophoblast-endothelium interaction studies. They reported that the co-culture of trophoblasts with endothelial cells significantly reduced the outgrowth and network formation of endothelial cells and induced endothelial cell apoptosis, shown by a significant upregulation of apoptosis marker (35). Trophoblast invasion is temporally and spatially regulated by autocrine (trophoblastic) and paracrine (uterine) factors as well as cell-to-cell and cell-to-matrix interactions (36). Trophoblast viability, migration and proliferation capacity as well as the expression of pro-apoptotic molecules like Fas-Ligand and tumor necrosis factor (TNF)-related apoptosis-inducing ligand have been shown to support SA remodeling (37–39). Also, the proteinase activity of EVTs influences their invasion capacity. Many studies demonstrate the importance of trophoblast cells for vascular remodeling by showing an association between insufficient trophoblast invasion leading to inadequate vascular remodeling and pregnancy complications, such as preeclampsia (PE) and intrauterine growth restriction (IUGR) (40, 41).

Interestingly, van der Heijden et al. postulated that the conceptus does not contribute to the initiation of uterine artery remodeling. They observed that the cellular processes of the remodeling, including artery lumen and cross-sectional area enlargement, reduced smoothelin expression, and increased VSMC proliferation is comparable in pseudopregnant and pregnant C57BL/6 mice (42). A limitation of this study is that the invasion of the trophoblasts is not as aggressive in mice as it is in humans and as consequence; SA remodeling is not fully comparable. In contrast to this particular study, other studies show that trophoblasts interact with maternal immune cells and this co-operation contributes to vascular changes. For example, the interaction of trophoblast HLA-G with uNKs impacts the maturation, proliferation, and mediator secretion of uNKs (43, 44) that in turn promote uterine vascular remodeling. Mediators of uNKs include chemokines, cytokines, proangiogenic factors, but also growth promoting factors like pleiotropin and osteoglycin. Hauk et al. reported with mouse models that the trophoblast-derived neuropeptide vasoactive intestinal peptide (VIP) is critical for MMP9 expression, migration and invasion capacities and that VIP-deficiency is associated with reduced Treg cell numbers at the feto-maternal interface (45). Further, the syncytiotrophoblast is reportedly able to communicate with maternal immune cells by secreting extracellular vesicles into the maternal circulation. These vesicles interact with monocytes, granulocytes, T cells and uNKs, influence their function, activation, and maturation. Extracellular vesicles from pre-eclamptic women influence immune cells differently when compared to extracellular vesicles from normal pregnant woman. For example, they fail to activate Tregs (46) and this might negatively influence the vascular remodeling during pregnancy.

### Uterine Natural Killer Cells

In humans, CD56^bright^ CD16^−^ uNKs can be found in the uterus at the beginning of each menstrual cycle as small agranular cells. Within the cycle, they grow and build numerous mediator-filled granules. Two days before menstruation, apoptosis of uNKs starts. In mice there is no change in the number of CD3^−^ CD122^+^ PAS^+^ uNKs during the murine estrous cycle, as they appear firstly after fertilization. During pregnancy in both, humans and mice, uNKs reach 70 % of all lymphocytes at the feto-maternal interface. This fact let to the assumption that they may play an important role in pregnancy. Cell numbers peak at midgestation, decline afterwards and are absent at term. In contrast to peripheral NKs (pNKs), uNKs show no cytotoxic activity and produce high amounts of cytokines, chemokines, and growth factors, for example transforming growth factor (TGF)-β, vascular endothelial growth factor (VEGF) and interferon (IFN)-γ (47, 48).

The differentiation, proliferation, activation, and survival of uNKs is regulated by sex hormones (49, 50) and the cytokine IL-15 (51–53). In addition, inhibitory or activating receptors regulate the function of uNKs. Many recent excellent works revealed the participation and importance of uNKs for the regulation of trophoblast invasion and SA remodeling (54–56). It is assumed that uNKs initiate SA remodeling before colonization by EVTs. In addition, uNKs communicate with trophoblast cells. This is possibly an explanation for the massive enrichment of uNKs near trophoblast cells. Trophoblasts express the non-classical HLA-E/G/C instead of the classical HLA-A/B as ligands for the inhibitory receptors of uNKs (57). As a result of the binding, uNKs may tolerate trophoblasts and support growth and migration of the fetal cells. uNKs may belong to the recently described innate lymphoid cell (ILC) family (58, 59) and more studies are needed to dissect which exact phenotype within this big family is pregnancy-protective. Furthermore, functional studies are needed to fully understand their participation in pregnancy-associated processes.

Mice that lack uNKs show impaired SA remodeling, characterized by thick walls and small vessel lumens, in contrast to control mice (60–63). Interestingly, many studies show that uNKs absence and associated abnormal SA remodeling does not affect progeny growth (61, 64, 65). In contrast, Fu et al. showed that the CD49a^+^Eomes^+^ subset of NK cells supports fetal growth in mice by the secretion of growth-promoting factors including pleiotrophin, osteoglycin and osteopontin. Absence of these cells resulted in fetal IUGR. Interestingly, significantly decreased percentages of CD49a^+^Eomes^+^ NKs as well as reduced levels of pleiotrophin, osteoglycin and osteopontin were found in first trimester decidua samples of patients suffering from recurrent spontaneous abortion compared to healthy donors (44).

Placental development is negatively affected in rodents that lack uNKs. An increase (61) or decrease (62, 63) in placental weight as well as markedly structural changes (65) were observed in recently published studies. Reasons for the different observations can derive from the fact that different mouse models were employed and different read outs were used as result. Ashkar et al. suggested that uNK-derived IFN-γ mainly contribute to uterine vascular modification at the feto-maternal interface in mice. The authors observed that females deficient for uNKs could initiate SA remodeling after receiving bone marrow from IFN-γ sufficient mice. Mice reconstituted with bone morrow from IFN-γ^−/−^ mice restored uNKs, but were not sufficient to initiate vascular remodeling (66). Human uNKs are able to secrete IFN-γ as well (67). IFN-γ derived by uNKs but also macrophage-derived TNF-α or IL-1β enhance IP-10 (CXCL10) and I-TAC (CXCL11) expression of decidual cells. In turn, these chemokines recruit CXCR3-expressing uNKs. The described regulation process is of clinical relevance, as its dysregulation is used for the prediction of preeclampsia, a disease that is characterized by an incomplete SA remodeling and reduced utero-placental blood flow that is associated with maternal hypertension (68). uNK-derived IFN-γ hinders an aberrant decidual cell MMP1, 3, and 9 expression and prevents thereby the occurrence of pre-eclampsia (69).

Choudhury et al. demonstrated that EVT-conditioned medium activates endothelial cells to secrete CCL14 and CXCL6 that in turn induce the chemotaxis of uNKs and macrophages. Both cell types express receptors for the mentioned chemokines (70). Based on these facts it is tempting to speculate that the crosstalk between fetal EVTs and maternal endothelial and immune cells including uNKs, supports the SA remodeling process that in turn ensures optimal fetal development.

### Macrophages

Macrophages reside within the decidua throughout pregnancy. M2 (alternatively activated)-like macrophages are more abundant than M1 (classically activated)-like macrophages in decidual tissue (71). Compared to M1, the M2 phenotype has a stronger pro-angiogenic potential due to higher expression of angiogenic factors (72, 73). In addition, human first trimester decidual macrophages express genes that are relevant for immunomodulation (74), like high levels of the anti-inflammatory IL-10 (75, 76). Additionally, it has been shown in an *in vitro* model of SA remodeling that human macrophages, isolated from early decidua, express genes for tissue remodeling and induce ECM breakdown of the ECM proteins laminin and fibronectin (77). Other experiments using first trimester placentation sites or 3D co-culture models proved an enrichment of macrophages in close proximity of invasive EVTs (78). Nevertheless, Lash et al. used invasion assays to show that early pregnancy macrophages do not to influence EVT invasion. Additionally, other authors utilized the chorionic plate artery (CPA) model and show that early decidual macrophages do not alter VSMC organization (77).

Apoptosis is an important process during uterine tissue remodeling and the invasion of the developing embryo during pregnancy. Clearance of the resulting apoptotic cells and cell debris presents a crucial event during uterine remodeling, as it is important to maintain tissue homeostasis and to protect the fetus (79). It has been shown that macrophages are important for the effective clearance of cell debris and apoptotic cells (77, 80) likewise trophoblasts, VSMCs, and endothelial cells. Uterine epithelial cells, that surround the blastocyst, undergo apoptosis during embryo implantation and need to be cleared by macrophages. Abrahams et al. postulated that this may explain the proximity of macrophages to EVTs at implantation sites (79).

Compared to those present at term pregnancies, decidual M2-like macrophages are reduced in preterm pregnancies and undergo an M1-like polarization during spontaneous term and preterm labor (71). Moreover, peripheral blood and placental/decidual tissue of preeclampic woman showed imbalanced IL-10 levels (81–85), that could be responsible for a defective M2 polarization of macrophages.

### Regulatory T Cells

It is has been proposed by us and many other authors, Tregs are important to induce and maintain immune tolerance toward the semi-allogeneic fetus (86–89). Additionally, several studies provided an association between diminished Treg frequency or disturbed activity and pregnancy complications (90–93).

An interesting recent study from Care et al. revealed that Tregs may also influence maternal vascular function. The authors observed dysregulated hemodynamics of the uterine artery after specific Treg depletion, shown by increased resistance and pulsatility indices as well as enhanced amount of active vasoconstrictors that derived in increased mean arterial pressure (94). In another study it has been shown that an conserved non-coding sequence 1 (CNS1)-dependent mechanism of extrathymic Treg cell differentiation is important for an effective SA remodeling (95). Besides, it was shown that the adoptive transfer of Tregs could reduce uterine perfusion pressure in a rat model of PE by decreasing levels of inflammatory mediators and reactive oxygen species (96). Other studies support the positive role of Tregs for vascular remodeling, and suggest that the mechanism rely on the ability of Tregs to modulate other decidual leucocytes like mast cells (MCs). Concretely, it was found that the transfer of Tregs into abortion-prone mice normalized early pregnancy angiogenesis that was associated with promoting the expansion of uterine mast cells (uMCs) (97). Altogether, it is tempting to speculate that the participation of Tregs and probably of other adaptive cells rely on their interaction with other cell types. An interesting study from 2017 points out that CD8^+^ T cell reconstitution in recombinase 1-deficient mice (Rag^−/−^, without T and B cells) before mating abrogates the resistance differences in vasculature that normally persist postpartum (98). Thus, adaptive immune cells overtake unsuspected roles during pregnancy that go beyond their classical function. The next challenge is to understand their participation using experimental models that have translational value.

### Uterine Mast Cells (uMCs)

uMCs are abundant in the reproductive tract of rodents and humans (99–101). They are subjected to the hormonal influence of progesterone and estrogen as they express hormone receptors (102). Numbers of uMCs oscillate within the cycle, being the highest at the receptive phase, and increasing during pregnancy (103).

It is well-established that MCs induce myometrium contractions that are important for the induction of birth (100, 104, 105). With the help of different MC-deficient mouse models (*Kit* mutation-dependent *K*it^W−sh/W−sh^ and *Kit*-independent Cpa3-Cre as well as Mcpt5-Cre^+^ R-DTA mice) it has been shown in the last years that MCs are also crucial for SA remodeling and fetal well-being (60, 63, 94, 100,103, 193). Interestingly, MC-deficient and NK-deficient mice show a comparable pregnancy phenotype, namely impaired SA remodeling. In our hands, the main difference between mouse models for NK- and MC-deficiency relies on the fetal weight outcome with the progeny of NK-deficient mice having normal weight and the progeny of MC- or MC/NK-deficient mice being growth restricted (64).

Poor fetal supply, often induced by insufficient vascular remodeling, can be recognized *v*ia ultrasound by an absent or reversed end diastolic flow and high resistance index of the umbilical artery. Those signs for neonatal IUGR could be found in MC/NK-deficient mice, that were growth restricted from midgestation onwards (106, 107). Interestingly, it was demonstrated that the absence of NKs was accompanied by an increased presence of MCs and *vice versa* (61) leading to the hypothesis that both cell populations work together to ensure a correct vascular modification. MCs are also able to communicate and influence the behavior of other immune cells like neutrophils, DCs, monocytes, macrophages, B and T cells (108–111). Whether the interplay of MCs with the mentioned cell types is relevant for pregnancy needs to be addressed in future studies.

The impressive consequence of MC absence in mouse models raises the question about the putative mediators involved in the remodeling processes at the feto-maternal interface. MC mediators are stored in cytoplasmic granules that can be released immediately upon stimulation. It has been shown that histamine supports ovulation and blastocyst implantation (112). Also chymases seems to play an important role for processes at the feto-maternal interface. The mouse chymase mast cell protease (Mcpt) 5 is expressed by both, uMCs and uNKs (64). *In vitro*, Mcpt5 mediated the apoptosis of VSMCs (64), an important feature of SA remodeling. Absence of Mcpt5^+^ cells in Mcpt5-Cre^+^ R-DTA mice derived in un-remodeled SAs and growth restricted progeny. Similarly, uMCs can be found at the human feto-maternal interface (113). MC-derived α-chymase CMA1 (the human Mcpt5 homolog) stimulated *ex vivo* the migration of human trophoblasts, a pre-requisite for an efficient SA remodeling process (64).

Chymases are able to convert pro-peptides of MMP2 and MMP9 into their active forms (114). MMPs are important regulators of trophoblast invasion and play an important role during SA remodeling (36). Further, chymases have been shown to degrade the ECM compounds (115). An extensive degradation of ECM compounds led to a loss of matrix survival signals and in turn to VSMCs apoptosis that favor SA remodeling. Additionally, chymases can directly inhibit the growth (116) or induce apoptosis (117) of VSMCs. Chymases are key enzymes of the renin-angiotensin-aldosteron-system (RAAS) that convert angiotensin (Ang) I to AngII independent of the angiotensin converting enzyme (118). Next to the systemic RAAS there exist tissue-specific local RAASs e.g., one in the placenta and another one in the decidua. A high local AngII concentration in the maternal part of the placenta and the resulting maternal-fetal AngII gradient contribute to trophoblast migration and invasion in early pregnancy (119). Additionally, utero-placental AngII is associated with an increased migration of VSMCs (120, 121), a process that is an important step effective SA remodeling (20). Another mediator that is necessary for MC function is the glycogen-binding protein galectin-1. *K*it^W−sh/W−sh^ mice have a reproductive impaired phenotype, including insufficient SA remodeling. The systemic reconstitution of mice with bone marrow-derived MCs from wildtype, but not Lgals1^−/−^ mice could normalize the remodeling of the arteries and contribute to fetal well-being (103), demonstrating a positive role for galectin-1 for MC function. We anticipate that future studies will contribute to an increasing understanding of the role of single MC mediators for pregnancy success. It is suggested that increased numbers or exacerbated activation of MCs is associated with human pregnancy pathologies like recurrent pregnancy losses (122) and PE (123, 124). The mechanisms underlying this phenomenon are not explored. That an absence of MCs derives in pregnancy pathologies, and an increased number of MCs is associated with pregnancy pathologies again supports the concept of perfectly regulated cellular processes as modulators of pregnancy.

## PE and IUGR—Adverse Consequences of Insufficient Uterine Remodeling

Insufficient remodeling of maternal uterine or vascular tissue reportedly leads to severe pregnancy disorders including miscarriage, preterm birth, PE or IUGR (125, 126). These pregnancy diseases dramatically impair maternal and fetal health or survival.

PE affects ~2–8 % of pregnancies worldwide. The multi-system disease is one of the most dangerous complication for both, mother and child with a high morbidity and mortality risk (127). Hallmarks are maternal hypertension that first manifested in pregnancy (≥140/90 mm Hg on at least 2 occasions, 6 h apart) and proteinuria (≥300 mg or greater in a 24 h urine, after 20 weeks of gestation) (128). Although the symptoms of the disease manifest in the second half of pregnancy, the pathogenesis is established during the first trimester, concretely during the time when SA remodeling takes place. Indeed, it has been shown that PE is associated with abnormal trophoblast invasion and inadequate SA remodeling during the first trimester (129, 130). This is thought to be a consequence of imbalanced immune cell numbers or activity. For example increased Th17 and impaired Treg activity, altered activation of macrophages, DCs, T cells, B cells, or NKs (131) were appointed as cause of impaired SA remodeling. To date, delivering the baby and removing of the placenta is the only effective treatment for PE. Here, a decision for the right time point of inducing the birth based on maternal health condition and fetal maturity must be taken.

Insufficient vascular remodeling can be associated with fetal IUGR (22). IUGR is defined as the failure of the neonate to reach its genetically determined growth potential and a weight under the 90th percentile compared to age-matched babies. IUGR occurs as consequence or independent of PE and results from inadequate blood supply due to impaired maternal SA remodeling (22). Besides, it can manifest due to an inadequate nutrition supply resulting from a failure of different placental transporter expression (132, 133). IUGR is associated with an increased risk of intrauterine death and a programming of diseases later in life such as hypertension, heart diseases, stroke, overweight, diabetes, metabolic syndrome or osteoporosis (134, 135).

**Figure 1:**
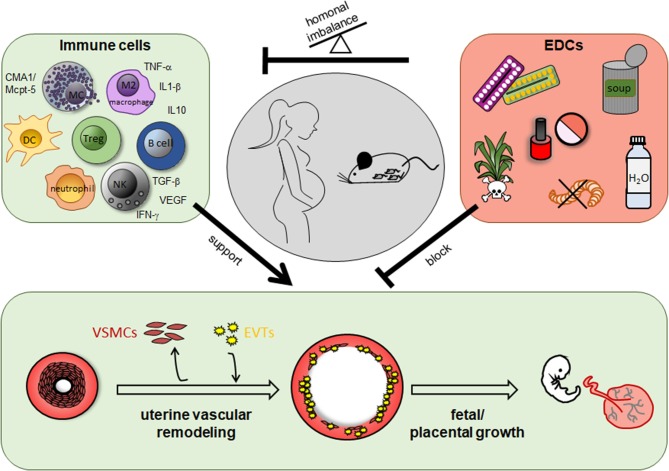
Role of immune cells and endocrine disrupting chemicals (EDCs) for uterine vascular remodeling during pregnancy. In humans and rodents including mice immune cells like uterine natural killer cells, mast cells, dendritic cells, T cells, especially regulatory T cells, B cells, macrophages and their secreted mediators support uterine vascular remodeling at the feto-maternal interface. EDCs are found in herbicides and pesticides as well as in many daily used products like cosmetics, plastic containers and bottles, food cans, and pharmacological drugs like contraceptive pills and many others. EDCs can impair uterine remodeling processes directly or indirectly due to a hormonal imbalance and in turn an altered immune cell number or function.

Additionally to the participation of immune cells in SA remodeling and hence, a role of a dysregulated immune response in abnormal SA remodeling, research of the last years emphasized the negative effects of man-made environmental substances in SA remodeling and pregnancy complications (Figure 1).

## Endocrine Disrupting Chemicals

The endocrine system is a network of glands and organs that produce and secrete hormones necessary for physiological processes including respiration, metabolism, reproduction, movement, sexual development, and growth. A finely modulated hormonal balance is indispensable for many pregnancy-related processes including time-dependent orchestrating of immune cells that contribute to vascular remodeling, as described earlier in the review. Hormonal imbalance of any kind can be associated with serious pregnancy complications like miscarriage (136, 137), PE (138, 139), preterm birth (140), or IUGR (141).

A large number of man-made or natural compounds, referred as endocrine disrupting chemicals (EDC), disturbs the endocrine system by interfering with biosynthesis, secretion, transport, metabolism, binding action, or elimination of hormones (defined by US Environmental Protection Agency). EDCs are able to bind, activate or inhibit hormone receptors (142) and can change hormone receptor expression levels in tissue (143).

Synthetic EDCs represent a heterogeneous class of molecules that includes plastics like bisphenol A (BPA) and plasticizers named phthalates, substances added to plastics to make them more flexible, durable, and transparent. Also pesticides like dichlorodiphenyltrichloroethane (DDT), fungicides, pharmaceutical agents e.g., diethylstilbestrol (DES), synthetic chemicals like polychlorinated biphenyls (PCBs), polybrominated biphenyls (PBBs), or dioxins, but also heavy metals among others belong to the group of EDCs (144). Due to their widespread usage, EDCs accumulate ubiquitously in our environment and enter the human body via different routes. These routes include inhalation, dermal via direct skin contact, or oral via food or beverages but also contaminated drinking water. Several results from animal studies and human epidemiological studies implicate EDCs as a significant treat to health and thus, a huge concern to public health. Adverse human health effects include metabolic, cardiovascular and behavioral disorders, but also hormone-related cancers (145–147). Also, reproductive failure in human and wildlife (148, 149) has been observed. Especially, people who are highly exposed to chemicals, pesticides, or fungicides at work are at particularly high risk for developing endocrine or reproductive abnormalities (144).

A challenge when working with EDCs is that these substances do not follow the typical dose-response dynamics, known for most chemicals in toxicological studies. In contrast, EDCs often show low dose effects or non-monotonic dose-response curves (150), defined as a non-linear relationship between dose and effect, like *U*-shaped or inverted-U shaped curves. Therefore, it is difficult to predict the response of an endocrine acting substance. Several EDCs are very stable and have a long half-life. This stability is beneficial for their industrial use, but raises the harmful effects from the use of the products for the environment and human health.

### BPA

Most studies that will be mentioned and discussed in the following part of this review, deal with BPA. BPA is one of the most produced and most studied EDC worldwide. The chemical is an organic compound consisting of two phenolic rings connected by a carbon carrying two methyl groups. It belongs to a subgroup of EDCs, the xenoestrogens, that exhibit estrogen-like properties by their ability to bind estrogen receptor (ER) α, ERβ, and the G-protein–coupled estrogen receptors (151–153). As many immune cells express ERs (154) they are highly receptive for the influence of xenoestrogens including BPA. Since the middle of the twentieth century, BPA is mainly used in the manufacturing of polycarbonate plastics and epoxy resins, both of which are used in a variety of daily used applications these days. For example hygiene and cleaning products, electronic devices, medical and dental devices, children toys, paints and coatings, cloth, food containers, plastic bottles, sport protection equipment, and even dental sealants. Under certain conditions BPA is released from the products. Especially high temperatures, e.g., placing plastic food containers in the microwave or the dishwasher, increase BPA release from the products (39). Also, when BPA is used as an additive e.g., for the coating of thermal paper or floor covering, BPA is not chemically bound and easily released from the products (155). BPA ending up in indoor air, dust, soil, wastewater, contact surfaces, food, and drinking water from which it enters the human body.

BPA is under scientific examination since many years. Based on the alarming findings, policies started to enforce restrictions on the manufacturing and use of BPA. Nevertheless, BPA is still present in uncountable daily used products. In 2017, BPA was identified as substances of very high concern (SVHC) under REACH (Registration, Evaluation and Authorisation of Chemicals) and is classificated today as a CMR (carcinogenic, mutagenic or toxic to reproduction) substance of category 1B, meaning BPA is a “presumed human reproductive toxicant based on animal studies.” Companies increasingly advertise for their BPA-free products (Websites European chemicals agency, Umweltbundesamt). What the consumers do not know is, that BPA is replaced by other bisphenols like bisphenol F or S that show comparable or even worse harmful developmental effects and are able to cross the placenta (156, 157). It was recently shown that the exposure of the population to BPA substitutes is almost ubiquitous (158).

Although the numbers of studies is increasing in the last years, the underlying mechanisms as to how exactly EDCs impair health are far from being understood and further research is urgently needed. Next, we will review recent findings concerning the influence of EDCs on reproductive parameters with special emphasis to the question if EDCs are able to influence vascular remodeling, placentation, or immune cell function.

## Effects of EDCs on Uterine Remodeling and Immune Cells

Exposure to EDCs in adults can have adverse health effects. During early development, when hormones play an important role, organisms are particularly sensitive to EDC exposure. Several EDCs including BPA are able to pass the placenta barrier and accumulate in placental tissue (159). Next to placental tissue, EDCs have been detected in the urine, cord blood, plasma, amniotic fluid, and breast milk of pregnant women and their developing fetuses (158, 160, 161). This represents a health problem for the mother and in more extent for the unborn, as many enzymes important for degrading dangerous substances are not expressed until birth.

### EDCs and Immune Cells

As discussed earlier in this review, a proper development and function of immune cells is indispensable for sufficient uterine remodeling processes during pregnancy. Based on that, we will give an insight into the current findings of the diverse influences of EDCs on immune cells in the following. We will mainly focus on the effects of immune cell phenotypes and functions, important for a successful pregnancy.

In mice, it has been shown that BPA exposure lead to phenotypic changes within distinct immune cell populations (162). Depending on the concentration and the route of administration, BPA decrease TNF-α and nitric oxide secretion of activated macrophages (163–165). Interestingly, BPA did not influence macrophage viability, but decreased the adherence ability of rat peritoneal macrophages (166). Taking into account that adhesion is the first step in the macrophages phagocytic process and that phagocytosis is important for the uterine remodeling process during pregnancy, as mentioned earlier in this review, BPA could in theory inhibit the remodeling process indirectly via macrophage inhibition.

Exposure of mice to BPA via drinking water for 4 weeks induced the production of the Th1 type cytokine IFN-γ and suppressed Th2 type IL-4 expression of CD4^+^ T cells (167). In contrast another study show an increase of Th2 polarization by an enhanced IL-4 production in antigen-activated T lymphocytes due to BPA exposure (168, 169). Further, Yoshino et al. demonstrated that prenatal fetal exposure to BPA upregulated Th1 but also Th2 responses in adulthood after immunization with hen egg lysozyme. This may explain the dramatic increase in allergic diseases over the last decades. Additionally, male but also female mice prenatally exposed to BPA had markedly increased numbers of splenic CD3^+^CD4^+^ and CD3^+^CD8^+^ cells (155). Number of Tregs was reduced in mice exposed to BPA either prenatally or in adulthood (169, 170). As discussed earlier, an imbalanced amount of immune cells can cause serious health consequences. Besides, also an imbalanced activity of immune cells due to decreased or increased receptor expression can have detrimental consequences for an organism. A study from 2012 point out that BPA, among other bisphenols, up-regulated HLA-class II, CD11c and CD86 in mouse bone-marrow-derived DCs (171). Further, an increase antibody production by B cells in mice was observed after BPA and DES exposure in mice (172, 173). O'Brien et al. show that the short-term exposure of both, BPA but also estradiol, at levels relevant to human exposure, enhances histamine release by primary bone marrow-derived MCs of mice. This constitutes yet another explanation for the increasing allergy prevalence. BPA additionally enhanced the release of cysteinyl leukotrienes. This was not ER-mediated (174). In contrast, the structure of the specific paraben, used as preservatives in cosmetic, medicines and food, determines if the EDC enhance, inhibit, or do not have any influence on the histamine release of rat peritoneal MCs (175). During the embryo stage of zebrafish, the EDCs 17β-estradiol, 17-α-ethynyestradiol (EE2), permethrin, atrazine, and nonylphenol significantly change innate immune-related gene transcription. This was shown by altered mRNA levels of TNF-α, IFN-γ, IL-1β, IL-8, CXCL-Clc, and CC-chemokine but also genes related to reactive oxygen species (176).

Synthetic EDCs also affect the development and the function of human immune cell populations (177). BPA exposure in human significantly increased in the proliferation of PBMC and modulated their cytokine production leading to a decrease in IL-10 and IL-13 expression. Additionally, BPA altered the phenotype of myeloid DCs by an increased CD1a, but decreased HLA-DR and CD86 expression (178). In contrast, it has been shown that BPA among other bisphenols decreased the expression of CD1a, CD80, CD86, and CD83 but increased the numbers of HLA-DR positive monocyte-derived DCs (179). In response to phthalates and the common EDCs nonylphenol and 4-octylphenol there was a modulation of DC cytokine expression (171, 180). Both of the latter EDCs but also BPA as well as bisphenol B and F interfere additionally with the differentiation of DCs (171).

Di-ethylhexyl-phthalate, dibutyl-phthalate, 4-tert-octylphenol, and BPA interfere with the TNF-α, IL-1β, and IL-8 cytokine secretion (181) and markedly reduce the phagocytosis ability of a human macrophage cell line (182) in an estrogen-mediated manner (181).

Contradictory results of studies may be explained the usage of very different concentrations or different administration routes or time points of the EDCs. Pharmacokinetic studies showed that the route of administration strongly influence the rate of metabolism of EDCs including BPA (183, 184) that in turn determine their concentrations in blood and tissues. For planning animal studies it should be kept in mind, that human beings are mainly exposed orally to BPA.

### EDCs and Uterine Remodeling During Pregnancy

A correct placentation is important to maintain maternal and fetal health not only throughout pregnancy, but also impacts the rest of their life. If EDCs interfere with the highly complex mechanisms of placental development or function and thereby impact pregnancy outcome, is under research.

In women that undergo *in vitro* fertilization (IVF), there is an inverse relationship between BPA urinary concentrations and estradiol levels as well as the total number of oocytes retrieved per cycle (185, 186). In addition, high urinary concentrations of BPA, parabens, and most phthalate metabolites were associated with lower probabilities of implantation, clinical pregnancy, and live birth after IVF (187). Phthalate exposure impacts human placental function by significantly modulating the expression of 93 critical placental genes like the epidermal growth factor receptor through methylation (188). Another study showed that high urinary concentrations of specific phthalate metabolites (mono(2-ethylhexyl) phthalate [MEHP], mono(2-ethyl-5-oxohexyl) phthalate [MEOHP], mono-*n*-butyl phthalate [MnBP), monoisobutyl phthalate [MiBP], and monobenzyl phthalate [MBzP]) were associated with a lower expression of the target genes reflecting trophoblast differentiation (189) that are in turn important for an efficient vascular remodeling during pregnancy. Moreover, pesticides disturb the hormonal network and the function of trophoblast cells as shown by reduced cell viability and altered hormone secretion and steroidogenesis gene expression in an ER-dependent manner (190).

Although there are many interesting studies about the effect of EDCs for human pregnancy, it is difficult to extrapolate the effect of single EDCs, as human are exposed to many EDCs at the same time. Studies using combined EDC exposure are needed in the future to fully understand the consequences on health.

In mice, short-term oral BPA-exposure (50 μg/kg bw/day) during early pregnancy provoked IUGR in more than half of the offspring from gd12 onwards as documented by ultrasound and fetal weight determination. Although velocity parameters of the uterine artery were normal, SA remodeling was impaired in BPA-exposed mice. This was shown by the fact that many VSMCs remain in the vessels walls in BPA-treated mice. Additionally, the SAs had increased wall thicknesses and increased wall-to-lumen ratios compared to control mice, both signs for insufficient remodeled SAs (191). Similar results were obtained by Ye et al. The authors demonstrated an abnormal vessel remodeling shown by increased retention of VSMCs and reduced vessel areas at the junctional zone of the placenta leading to PE-like features including hypertension. Additionally, they found a decreased expression of MMP2 and 9and an enhanced expression of TIMP1 and−2 in BPA-treated mice that might be the reason for an impaired invasion of the trophoblast cells leading to insufficient remodeled vessels (192). Indeed, *in vitro* it was shown that BPA impairs trophoblast invasion (192). In these mouse studies it was not clarified if BPA influenced other cells next to trophoblasts. Whereas, uNK and uMCs numbers were not affected (191), a possible influence of the EDC on the activity of the immune cells was not analyzed and is an interesting question for further research. Morphological changes in the placentas of BPA-treated mice, including a smaller labyrinthine zone, narrow intervillous spaces, and degenerative changes in the trophoblastic giant cells and spongiotrophoblast layers were reported. This was associated with a decreased number and weight of embryos (193).

Next to BPA, other EDCs have been analyzed and the results obtained strongly suggest a disturbance of uterine vascular remodeling by EDCs. PCB-exposure of the mink let to degenerated placentas, characterized by vascular lesions in the labyrinthine zones, degeneration of endothelial and trophoblast cells, and hemorrhage. This was associated with fetal growth restriction and death (194). EE2, that is massively used as a compound in contraceptive pills, accumulate in the environment, open waters and enters the human food chain. A recent study shows that oral application of EE2 in mice during early pregnancy leads to reproductive impairments. In a high concentration (5 μg/kg bodyweight (bw)/day) EE2 leads to death of all fetuses in 80 % of the animals at midpregnancy. In contrast, a lower concentration (5 ng/kg bw/day) did not affect fetal survival but was clearly associated with impaired SA remodeling and abnormal increased fetal and placental growth (195). Thus, the impact to fetal and placental growth is different depending on the nature of the EDC.

As earlier mentioned in this review, most EDCs follow non-monotonic dose-response curves. This fact makes it difficult to draw conclusions from EDCs research results. Nevertheless, there is evidence that EDCs do not only exert a direct negative effect on vascular remodeling during pregnancy, but also an indirect effect by their ability to alter immune cell function. Many effects followed by EDC exposure, especially BPA, were not only dose-, but also sex-specific (196). The interesting field of phenotypic gender differences by EDC exposure is still underrepresented and opens potentially new interesting research fields. Also, the epigenetic effect of EDCs is an interesting topic for more detailed research and is not contemplated in this review.

Taken together, the results of numerous research studies clearly demonstrate that EDCs greatly impact the immune system and uterine remodeling during pregnancy that may have harmful effects for the mother and even more for the developing fetus. It is important to mention that most studies investigate the health effects of single EDCs exposure, even though exposures do occur as chemical mixtures of EDCs that most probably impact health quite different and unpredictable. Avoiding unnecessary EDCs-containing products, especially during the critical time of fetal development during pregnancy, may help avoiding harmful health outcomes.

## Conclusions

The present review aimed to summarize long-term established and brand-new research results concerning the indispensable role of innate immune cells for an effective uterine remodeling process. Additionally, the manuscript visualizes serious short- and long-term health consequences for mothers and children followed by an ineffective uterine remodeling process. An advanced understanding of mechanisms and disruptors of uterine remodeling helps to identify factors involved in physiological or pathological pregnancies. The long-term goal of research is to develop novel therapeutic options against pregnancy disorders, to elucidate and reduce potential negative effects on unborn life, and to improve *in-vitro*-fertilization techniques.

Moreover, the review focused on the reproductive health consequences of EDCs that accumulate non-stop in our environment and are impossible to eliminate or neutralize. We are convinced that research has an impact on the understanding of diseases and the potential to improve health care worldwide.

## Author Contributions

NM reviewed the literature and wrote the manuscript. AZ reviewed the manuscript.

### Conflict of Interest

The authors declare that the research was conducted in the absence of any commercial or financial relationships that could be construed as a potential conflict of interest.
